# Vaccination with a replication-defective cytomegalovirus vaccine elicits a glycoprotein B-specific monoclonal antibody repertoire distinct from natural infection

**DOI:** 10.1038/s41541-023-00749-0

**Published:** 2023-10-10

**Authors:** Sarah M. Valencia, Eric Rochat, Melissa J. Harnois, Maria Dennis, Helen S. Webster, Bhavna Hora, Amit Kumar, Hsuan-Yuan (Sherry) Wang, Leike Li, Daniel Freed, Ningyan Zhang, Zhiqiang An, Dai Wang, Sallie R. Permar

**Affiliations:** 1 Duke University Medical Center, Duke Human Vaccine Institute, Durham, NC 27710 USA; 2 Department of Pediatrics, Weill Cornell Medicine, New York, NY 10065 USA; 3 Texas Therapeutics Institute, Brown Foundation Institute of Molecular Medicine, The University of Texas Health Science Center at Houston, Houston, TX 77030 USA; 4 Merck & Co., Inc., Rahway, NJ USA

**Keywords:** Immunology, Vaccines

## Abstract

Human Cytomegalovirus (HCMV) is the leading infectious congenital infection globally and the most common viral infection in transplant recipients, therefore identifying a vaccine for HCMV is a top priority. Humoral immunity is a correlate of protection for HCMV infection. The most effective vaccine tested to date, which achieved 50% reduction in acquisition of HCMV, was comprised of the glycoprotein B protein given with an oil-in-water emulsion adjuvant MF59. We characterize gB-specific monoclonal antibodies isolated from individuals vaccinated with a disabled infectious single cycle (DISC) CMV vaccine, V160, and compare these to the gB-specific monoclonal antibody repertoire isolated from naturally-infected individuals. We find that vaccination with V160 resulted in gB-specific antibodies that bound homogenously to gB expressed on the surface of a cell in contrast to antibodies isolated from natural infection which variably bound to cell-associated gB. Vaccination resulted in a similar breadth of gB-specific antibodies, with binding profile to gB genotypes 1–5 comparable to that of natural infection. Few gB-specific neutralizing antibodies were isolated from V160 vaccinees and fewer antibodies had identifiable gB antigenic domain specificity compared to that of naturally-infected individuals. We also show that glycosylation of gB residue N73 may shield binding of gB-specific antibodies.

## Introduction

Human Cytomegalovirus (HCMV) is a pathogen that has coevolved with humans for millions of years and over half of the human population is infected. Infection in most individuals is asymptomatic, however immunocompromised individuals can have significant complications associated with HCMV infection. It is the most common viral infection in transplant recipients and can lead to disease and organ rejection^1^. HCMV is also the most common congenital infection, which can have significant long-term neurologic consequences for the infected fetus^2^. In its coevolution with the human immune system, the virus has acquired numerous immune evasion strategies. In fact, natural immunity does not completely protect from reinfection, though prior immunity can significantly reduce risk of disease associated with HCMV after transplantation and the risk of congenital infection^3,4^.

While cellular immunity is critical to virologic control of HCMV, humoral immunity has been implicated as an immune correlate of protection against HCMV acquisition and congenital transmission^5–7^. Glycoprotein complex gH/gL/UL128/UL130/UL131A (pentameric complex, PC)^8^, gH/gL/gO, and glycoprotein B (gB) are the main glycoproteins on the surface of the HCMV virion that mediate cell entry. gB is the primary viral fusion protein and has been heavily studied for use as a vaccine immunogen due to its abundance and immunogenicity. In fact, a gB protein-based vaccine adjuvanted with the squalene adjuvant MF59 was approximately 50% effective at preventing HCMV acquisition and/or reducing viremia in phase 2 clinical studies^9–11^. Until recently, the prefusion structure of the gB fusogen has remained elusive and to date there are still unstructured domains on the protein^12^. Designing a gB immunogen that would present the optimal conformational epitopes to elicit protective immunity is currently hindered by a lack of understanding of which epitopes are critical to elicit antibody responses that prevent HCMV acquisition and/or disease.

Recent studies have indicated that both neutralizing and non-neutralizing antibody responses could be important to protection against HCMV^7,13^. In follow up studies of the partially protective gB/MF59 vaccine clinical trial, we determined that IgG binding to cell-associated conformation of gB, but not the soluble gB antigen, was a correlate of protection against HCMV acquisition in gB/MF59 vaccinees^5^, indicating the importance of antibody binding to the native-like gB conformation in their ability to mediate protection and the need to further define antibodies that recognize this conformation of gB to aid in immunogen design. Interestingly, the disabled infectious single cycle (DISC) V160 vaccine also achieved partial efficacy against HCMV acquisition with a vaccine efficacy of 42% in a phase 2 trial^14^, indicating that this vaccine which displayed all HCMV glycoproteins in a single-round infection was similar to that of the gB immunogen alone. Studies of adding the PC to gB immunogens also did not achieve vaccine efficacy in nonhuman primate models^15^. The gB-specific antibody response remain the only identified vaccine-elicited immune correlate of protection against HCMV acquisition. Therefore, improving the quality of this response remains a tenant of HCMV vaccine development.

Glycosylation of surface glycoproteins shapes the antibody recognition of many viruses including influenza^16^ and HIV^17^. Epitopes on viral glycoproteins that make the virus sensitive to antibody-mediated neutralization or non-neutralizing antibody effector functions can have nearby glycosylation sites that result in glycan shielding and immune evasion by the virus. HCMV gB is uniquely heavily glycosylated^18^ with 18 predicted glycosylation sites. Domain I and II are the most heavily glycosylated regions followed by AD2 and the furin cleavage site^19^. The N terminal AD-2 region is one of the last unstructured gB domains and contains three N linked glycosylation sites; asparagine (N) 68, 73, and 85. AD-2 site I is a target of potent neutralizing antibodies, predicted to correlate with reduced viremia^20^. Yet, antibodies directed against AD-2 site I are detected in only 50% of seropositive individuals.

In the present study, we sought to define the gB-specific monoclonal antibody repertoire elicited in individuals exposed to gB expressed on the surface of a cell or virion, including individuals vaccinated with an HCMV DISC virus vaccine, V160^21^, and compare it to those who are naturally infected with HCMV^22^. Further, we aimed to determine if there are interactions with glycans on the gB protein required for their binding and functionality. We hypothesized that HCMV DISC vaccines and natural infection would elicit gB-specific antibodies directed towards cell-associated gB that were both neutralizing and non-neutralizing, providing the opportunity to define epitope targets of antibodies with desirable anti-viral functions. Moreover, we hypothesized that the gB-specific antibody binding and function may be dependent on engagement with N-linked glycans. Further understanding of the antibodies that can bind to vulnerable epitopes on the main viral fusogen and mediate important anti-viral functions will be critical for enhancing the efficacy of current generation HCMV vaccines.

## Results

### Epitope domain mapping of gB-specific monoclonal antibodies isolated from V160 vaccinated individuals

**Fig. 1:**
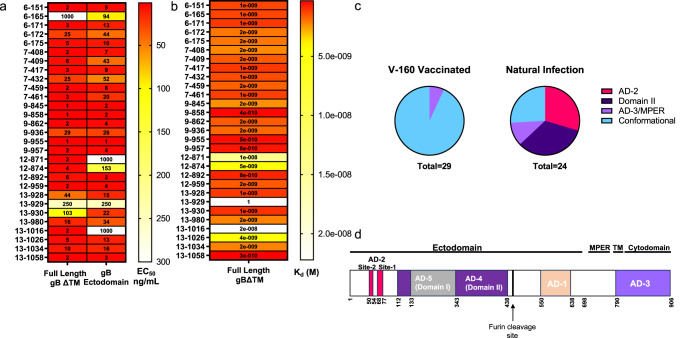
Epitope domain mapping of gB-specific monoclonal antibodies isolated from V160 vaccinated individuals. **a** Heat map of gB-specific monoclonal antibodies binding represented as EC50 ng/mL to FL gB Δ transmembrane domain (TM) or gB ectodomain. **b** Heat map of binding kinetics of gB-specific monoclonal antibodies measured by SPR (K_d_ (M)) to FL gB ΔTM. **c** Pi chart representing number of gB-specific monoclonal antibodies binding to specific gB domains measured by binding antibody multiplex assay. Out of either 24 total monoclonal antibodies from naturally infected (binding data from ref. ^22^) or 29 monoclonal antibodies from V-160-vaccinated individuals (binding data in Supplementary Fig. 1). AD-3/MPER indicates binding to full length gB but not gB ectodomain. Conformational binding indicates binding to full length gB and gB ectodomain, but no other domain. **d** Diagram of gB antigenic domains.

The HCMV-specific memory B cell repertoire was analyzed in 6 HCMV-naive individuals post vaccination with V160 vaccine. As previously described, memory B cells were isolated from circulating mononuclear cells. Memory B cells were enriched using EasySep memory B cell kit followed by plating in 384 well plate with feeder cells. Supernatant from expanded memory B cells were then screened for binding to HCMV whole virion and neutralization on ARPE-19 cells^23^. B cells producing monoclonal antibodies that bound to HCMV whole virions and were neutralizing on ARPE-19 cells were selected for further characterization. Non-neutralizing antibodies were also selected, however the number of non-neutralizing antibodies that were characterized were reduced through random selection to thirty percent of total number to match the number of neutralizing antibodies. Given the partial success of gB antigen vaccines, we further characterized the 29 gB-specific monoclonal antibodies from this subset of V160-elicited HCMV-specific monoclonal antibodies. Overall, the binding strength, measured by ELISA, and avidity, measured by SPR, of these vaccine-elicited gB-specific monoclonal antibodies for full length gB with the transmembrane domain deleted (gBΔTM) and gB ectodomain (both gB-1 genotype from Towne strain) were uniformly high magnitude (Fig. 1 and Supplementary Table 1), similar to the binding profile described for gB-specific monoclonal antibodies isolated from naturally-infected individuals^22^. The exception was one V160-elicited gB-specific antibody, 13–929, which displayed negligible binding to both full length gBΔTM and ectodomain gB, however demonstrated binding in the whole virion ELISA screen, as well as cell associated gB.

HCMV gB has 5 defined antigenic domains^19^. We used a previously established binding multiplex assay (BAMA)^24^ to determine the specific gB domain that the V160 vaccine-elicited gB-specific monoclonal antibodies were directed against. gB domains analyzed included gB AD-1, Domain I (AD-5), and Domain II (AD-4), all based on Towne strain gB (genotype 1), and gB linear domains AD-2 Site 1 and AD-2 Site 2. Binding to the AD3/MPER domain was defined based on the antibody binding to full length gBΔTM, but not gB ectodomain based on ELISA. Interestingly, none of the V160 gB-specific monoclonal antibodies tested at 30 µg/mL and 100 µg/mL concentration mapped to a specific gB antigenic domain (Supplementary Fig. 1 antibodies tested at 30 µg/mL). In contrast, 14 of 24 gB-specific monoclonal antibodies previously isolated from naturally-infected individuals tested at 30 µg/mL mapped to antigenic domains including Domain II (*n* = 9) and AD-2 site 1 and/or 2 (*n* = 8)^22^. A subset of previously-described^22^ gB-specific monoclonal antibodies isolated from naturally infected individuals that had defined domain specificity were run with this panel of gB-specific monoclonal antibodies isolated from V160 vaccinated individuals as a control.

### HCMV gB-specific monoclonal antibodies isolated from V160-vaccinated individuals and naturally infected individuals have similar gB genotype binding breadth

**Fig. 2:**
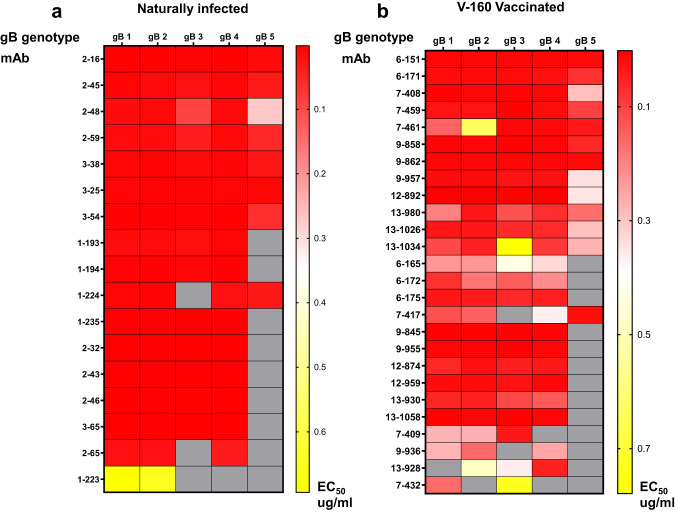
HCMV gB-specific monoclonal antibodies isolated from V160-vaccinated individuals and naturally-infected individuals have similar gB genotype binding breadth. ELISA measuring binding of gB-specific monoclonal antibodies from naturally infected individuals (**a**) or V160 vaccinated individuals (**b**) to gB ectodomain genotypes 1–5 with an EC50 < 1 ug/ml.

HCMV has been described to have high population diversity with a high number of strains sequenced from clinical samples often defined by glycoprotein genotypes^25^. Interestingly, mixed strain infections have been associated with increased disease progression specifically in stem cell transplant patients^25^. Further, gB genotypes can be distinctly recognized by gB-specific monoclonal antibodies^22^ and it is possible that infection by distinct gB genotype strains contributed to the partial efficacy of the gB/MF59 vaccine^26^. gB has five specific genotypes (alignment in Supplementary Fig. 6) and the V160 vaccine was designed based on a gB 2 genotype strain AD169. We analyzed the gB-specific monoclonal antibodies to see if a single gB genotype DISC vaccine could elicit similar levels of gB breadth compared to a natural infection. The genotype breadth of the gB-specific monoclonal antibodies was measured by ELISA. Only antibodies that bound gB ectodomain with an EC50 < 1 µg/ml were included in the analysis against all 5 gB ectodomain genotypes. Monoclonal antibodies isolated from V160-vaccinated individuals compared to that of naturally-infected individuals resulted in similar levels of gB genotype breadth (Fig. 2). While gB genotype 1–4 binding was common, yet variable, between gB-specific monoclonal antibodies, both vaccinated and naturally-infected individuals had fewer gB-specific monoclonal antibodies that bound genotype 5.

### V160-vaccinated individuals produced gB-specific monoclonal antibodies with homogenous high binding to cell-associated gB

**Fig. 3:**
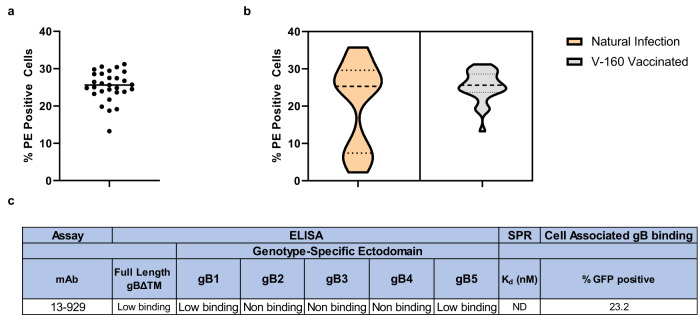
V160-vaccinated individuals produced gB-specific monoclonal antibodies with homogenous high binding to cell-associated gB. **a** Cell associated binding of gB-specific mAbs from V160-vaccinated individuals. **b** Violin plot representing gB cell associate binding from gB mAbs isolated from naturally infected^22^ or V-160 vaccinated individuals. Data from naturally infected mAbs is from ref. ^22^. Dashed line represents median binding and dotted line represents quartile binding within each cohort. **c** mAb 13–929 uniquely binds to cell-associated gB, but poorly to FL gBΔTM and gB ectodomain genotype 1–5.

Antibody binding to the cell-associated conformation of gB has been identified as a correlate of protection against HCMV acquisition in the gB/MF59 vaccine trials^5^. All gB-specific monoclonal antibodies isolated from V160-vaccinated individuals bound homogeneously to cell-associated gB (Fig. 3a). In contrast, gB-specific monoclonal antibodies from naturally-infected individuals had two distinct populations of binding to cell associated gB, a high and low binding population(Fig. 3b)^22^. Further, among the gB-specific antibodies isolated from V160-vaccinated individuals, we identified a unique gB-specific monoclonal antibody 13–929 that bound potently to the cell-associated gB conformation yet demonstrated only poor binding to soluble full length gBΔTM and no detectable binding to any of the five soluble gB genotype ectodomains (Figs. 3c, 1a and Supplementary Fig. 2).

### Glycosylation of N73 in the AD-2 site 1 region shields gB-specific antibody binding

**Fig. 4:**
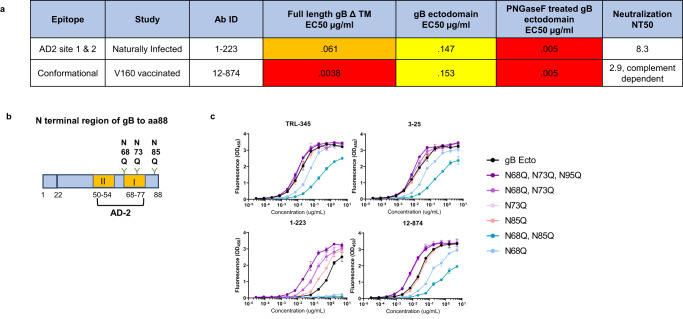
Glycosylation of N73 in the AD-2 site 1 region shields gB antibody binding. **a** Table of gB-specific monoclonal antibody binding to FL gB ΔTM, gB ectodomain or gB ectodomain treated with PNGase F to remove N linked glycosylation and neutralizing titer NT50 (Neutralization titer is described in ref. ^23^). **b** Figure representing N terminal gB indicating where the asparagines were mutated to glutamine by site directed mutagenisis. **c** ELISA showing binding of AD-2 site 1 potently neutralizing specific mAbs (TRL-345 and 3–25), 1–223, and 12–874 to gB ectodomain or gB ectodomain with asparagine mutated to glutamine at the indicated amino acid.

We first investigated whether glycosylation globally contributed to binding of gB-specific monoclonal antibodies by treating the gB ectodomain protein with PNGaseF to remove N-linked glycosylation and comparing the vaccine and naturally elicited gB-specific monoclonal antibody binding before and after deglycosylation (Supplementary Fig. 3). Interestingly, two monoclonal antibodies, 1–223 and 12–874, isolated from a naturally-infected individual and a V160-vaccinated individual, respectively, had a 30-fold increase in binding to gB ectodomain treated with PNGaseF compared to untreated (Fig. 4). In addition, one of these monoclonal antibodies isolated from a naturally-infected individual, 1–223, mapped to bind to both gB AD-2 site 1 and 2 in BAMA. Thus, we used site-directed mutagenesis to mutate the glycosylation sites in AD-2 via an asparagine (N) to glutamine (Q) mutation, an amino acid substitution which also contains an amide side chain but is not glycosylated. Further, we made a triple mutant (N68Q, N73Q, and N95Q), two double mutants (N68Q and N73Q) or (N68Q and N85Q) and three single mutants N73Q, N85Q, and N68Q. AD-2 site 1-specific and potently-neutralizing monoclonal antibodies TRL345 and 3–25^27,28^ bound equally well to all gB glycan mutants suggesting that glycosylation does not block binding to the conserved, linear neutralizing domain on AD2 site 1 and is not contributing to the potent neutralization mediated by these antibodies (Cytogam binding is shown to all gB mutants in Supplementary Fig. 4). However, binding was increased for the poorly-neutralizing gB-specific monoclonal antibodies 1–223 and 12–874 to the triple mutant (N68Q,N73Q,N85Q), the double mutant (N68Q and N73Q), as well as the single N73Q mutant (Fig. 4). These data suggest that glycosylation of the N73 asparagine shields antibody binding to the AD-2 region. Our results suggest that both DISC vaccination and infection can elicit gB AD-2-specific antibodies with weak binding and neutralizing activity that are susceptible to gB glycan shielding in the AD2 region.

## Discussion

Thus far, HCMV vaccine candidates have only achieved partial protection against acquisition of HCMV in seronegative individuals and did not reach high enough efficacy to move forward towards licensure. The most well studied vaccine, gB/MF59, indicated that gB could be an effective vaccine target capable of eliciting anti-viral antibody effector functions such as antibody-dependent cellular phagocytosis and binding to cell-associated gB, the latter identified as a correlate of protection. The addition of other glycoprotein complexes to the HCMV vaccine, such as the pentameric complex, while a promising approach, has not yet demonstrated increased vaccine efficacy^29^. The V160 DISC vaccine showed 42% reduction of infection in individuals vaccinated with 3 doses in a recent phase II trial^14^. Our study leveraged the opportunity to evaluate the monoclonal antibody repertoire elicited by vaccination with a replication defective vaccine virus against the main viral fusogen, gB, compared to that elicited by natural infection. The global HCMV-specific B cell repertoire in V160 vaccinated individuals was previously examined^23^ and was found to contain specificity for multiple glycoprotein targets, including the pentameric complex and also contain functional neutralizing antibodies.

Consistent with our previous study including naturally infected individuals, we found that vaccine-elicited gB-specific mAbs have high affinity and avidity to gB. However, none of the gB-specific mAbs had high neutralizing activity^30^. We also measured Fc-mediated ADCP activity for the DISC vaccine-elicited mAbs (Supplementary Fig. 5) and none of the 29 mAbs demonstrated ADCP activity compared to 2 of 27 the mAbs isolated from naturally-infected individuals. One caveat for all mAbs included in this study is that they were cloned into an IgG1 backbone. Some of the mAbs natively might have been IgG3 which may have increased the number of antibodies with ADCP activity. We then mapped the domain specificity of these antibodies and demonstrated that none of the 29 vaccine-elicited gB-specific mAbs had identified domain-specificity, unlike the AD-2 site 1 and Domain II specific mAbs identified in the gB-specific B cell repertoire isolated from naturally-infected individuals. These results suggest that while the V160 vaccine elicited B cell responses to multiple HCMV glycoprotein targets^30^, the gB-specific B cell responses less frequently had detectable neutralizing and non-neutralizing function compared to that of naturally-infected individuals. Potentially, presentation of a complex immunogen such as whole virus and/or using a replication defective virus which has a short replication cycle compared to natural infection might limit the development of highly functional antibodies to a specific protein such as gB.

Circulation of diverse HCMV variants could result in a less effective humoral immune response^31,32^ and there is suggestion that HCMV variants may lead to an increased susceptibility to reinfection^25^. We measured the gB variant-specific binding breadth of gB-specific mAbs from the gB genotype 2-based DISC virus vaccinees and from naturally infected individuals against gB genotypes 1–5. While there is equal magnitude binding of gB-specific mAbs isolated from naturally infected and DISC-vaccinated individuals against gB genotype 1–4, there was a reduction in binding to gB-5 by gB-specific mAbs from both populations. Since gB-5 has low representation in the population these results are not unexpected, however the 5 genotypes have high levels of homology ~95%^25^. The reduction in gB-specific mAbs recognition of gB 5 suggests that small mutations within gB are possible that would result in a less effective antibody repertoire. Thus, it will be important to look at structural differences of gB and its recognition by functional antibodies that occur with naturally occurring amino acid mutations in circulating populations.

We predicted that presentation of gB in the context of a replication defective virus would elicit an antibody repertoire that binds cell-associated gB. We expect that cell-associated gB is a more native conformation of gB compared to soluble gB. All gB specific mAbs isolated from DISC vaccinated individuals bound well to cell-associated gB compared to the distinct populations of low and high-binding gB-specific mAbs to cell-associated gB isolated from naturally infected individuals^22^. Uniquely, we found a gB-specific mAb 13–929 that bound specifically to cell-associated gB, but poorly to soluble gB. This is evidence that there is a unique epitope that is presented on cell-associated gB, but not on soluble gB. Antibody 13–929 was identified in the original binding to whole virion screen but removed from the Li et al.^23^ paper after they did not find a specific glycoprotein complex that this antibody bound to. Since 13% of the virion-binding mAbs isolated were gB-specific, cell-associated gB binding could be used to screen for functional gB-specific mAbs within the B cell repertoire of vaccinated or naturally infected individuals. This may be important because antibody responses against cell-associated gB, but not soluble gB, correlated with protection in the vaccine study^5^.

Finally, we looked at the effect of glycan shielding on binding of vaccine and natural infection-elicited mAbs to the gB protein. In a screen for glycan-dependence of gB-specific mAbs using removal of N linked glycans with PNGaseF, we identified two mAbs that bound 30-fold higher to gB ectodomain after removal of glycans. Since 1–223 was identified as specific to the AD-2 region, which is a heavily glycosylated, we used site directed mutagenesis to determine if glycosylation of AD-2 was shielding binding of the two mAbs. Through site directed mutagenesis of glycosylation sites in the AD-2 region of gB we show that N73 is a potential site of glycan shielding on gB. While the highly potent neutralizing mAbs 3–25 and TRL345 which bind specifically to AD-2 site 1 are not affected by these glycosylation mutations, our data suggests that there is a portion of antibodies with low-level neutralizing activity that are directed to a site on AD-2 that is shielded by glycosylation.

Modifying the gB immunogen will likely be a critical parameter to improve humoral immunity elicited by vaccination. Structure based design of viral fusogens has shown efficacy with other viruses such as RSV and HIV^33,34^. With an expanding breadth of research measuring correlates of protection for HCMV vaccines, specific gB antigenic targets have come to the forefront as significant, such as AD-2 site 1 which harbors a site specific for highly neutralizing antibodies^20,28^, antibodies that bind to cell-associated gB^5^ and antibodies that mediate Fc-mediated effector function, such as ADCP have also have been associated with protection^35^. Recently, the challenging prefusion gB structure has been published^12^, which will advance identification of prefusion gB antibodies and development of prefusion gB immunogens. While sterilizing immunity is a high bar for HCMV vaccines, humoral immunity has shown to be associated with prevention of congenital HCMV and reduction in HCMV disease in transplant patients, suggesting that a vaccine that provides partially-protective immunity against infection would significantly reduce the disease and burden of HCMV^35^.

## Methods

### Human subjects and isolation of gB-specific monoclonal antibodies

This study aimed to isolate and analyze the specificity and function of the HCMV vaccine (V160) elicited monoclonal antibodies. It is a sub-study of a previous phase I clinical trial (NCT01986010, registered on 18 November 2013). The V160 vaccine is a disabled live attenuated virus based on the AD169 virus (gB 2 genotype) that contains a repaired pentameric complex, recently tested in a phase 2 study^14^. HCMV seronegative women were vaccinated with three dose regimen of the V160 vaccine. Six random individuals are included in this study (3 vaccinated intramuscular with 30 U V160 and 3 vaccinated intradermal with 30 U V160). We also enrolled three HCMV-seropositive subjects for positive control (enrolled in University of Texas Medical Branch)^23^. Healthy subjects eligible for inclusion in this sub-study were over 18 years of age with body weight >50 kg and body mass index 19–32 kg/m^2^. The trial was performed in conformance with standards of Good Clinical Practice. The protocol was reviewed and approved by the Western Institutional Review Board, Inc., and the Institutional Review Board of the University of Texas Medical Branch. Subjects provided written informed consent before participation. Memory B culture were isolated from plasma PBMC and supernatant screening protocol outlined in Li et al.^23^ paper. B cell supernatants were screened by HCMV virion binding and neutralizing capabilities against AD169r-GFP virus infection of ARPE-19 cells. 30% of non-neutralizing positive hits were included for further screening. Antibodies were then cloned and purified. Specific glycoprotein binding specificity was determined by ELISA. 29 gB-Specific monoclonal antibodies were identified.

### Enzyme Linked Immunosorbent Assay (ELISA) to assess IgG binding to HCMV gB

Monoclonal antibody binding to gB was measured against full length gB protein (Sanofi), gB ectodomain representing gB genotypes 1–5; gB-1 from Towne strain (GenBank accession number: ACM48044.1; gB-2 from AD169 strain, GenBank accession number: DAA00160.1), gB-3 (Toledo strain, GenBank accession number: ADD39116.1), gB-4 (C194 strain, GenBank accession number: AAA45925), and gB-5 (saliva isolate, GenBank accession number: AZB53144), gB ectodomain genotype 1 from Towne strain deglycosylated with PNGaseF, and biotinylated linear gB AD-2 (biotin-NETIYNTTLKYGD). For gB genotype (1–5) ELISAs, hyperimmune globulin (HIG, Cytogam) was diluted 1:1000 and run in a 3-fold dilution series as a positive control and all mAbs were run in parallel at a starting concentration of 5 ug/ml in a 3-fold dilution series. For the gB ectodomain mutant proteins and gB-AD2 peptide ELISAs, TRL-345 and 3–25 mAbs were used as positive controls; naturally infected mAbs and controls were plated at a starting concentration of 10 ug/ml and run in a 3-fold dilution series. All proteins and peptides were diluted in 0.1 M NaHCO_3_ coating buffer and plated at either 1 µg/mL (gB genotype proteins) or 2 µg/ml (gB mutant proteins and gB-AD2 peptides) in 384-well plates. Plates were incubated at 4 °C overnight, washed, blocked for 1 h, and mAbs were added. After a 1 h incubation at RT (gB proteins) or overnight incubation at 4 °C (gB mutant peptides and gB AD-2), plates were washed twice and goat anti-human HRP-conjugated IgG (Jackson ImmunoResearch Cat.No.109–035–003) secondary Ab was diluted 1:5000 and added for 2 h. Plates were developed during a 5–10 min incubation with SureBlue substrate (VWR), followed by stop solution (VWR). Plates were read at 450 nm on a Spectramax plate reader (ThermoScientific).

### Binding antibody multiplex assay (BAMA)

Epitope mapping of plasma samples was achieved through BAMA. Carboxylated fluorescent beads (Luminex) were covalently coupled to purified HCMV antigens then incubated with plasma samples in assay diluent (PBS, 5% normal goat serum, 0.05% Tween-20, and 1% milk). Plasma samples were measured for binding against gB proteins AD-1 (myBiosource, AA 549–645), gB Domain I (produced in-house, AA 133–343, and both the 3′ avidin/polyhistidine tags omitted due to hypothesized steric hinderance), gB Domain II (produced in-house, AA 112–438 + 343–438, joined with the flexible linker Ile-Ala-Gly-Ser-Gly,), gB Domains I + II, and gB ectodomain at a 1:500 dilution. All gB proteins are based on the Towne strain gB (genotype 1). Plasma dilutions were determined to be within the linear range of the assay. HCMV glycoprotein–specific antibody binding was detected with phycoerythrin-conjugated goat anti-human IgG (2 µg/mL, Southern Biotech Cat. No.2040–09). Beads were washed and acquired on a Bio-Plex 200 (Bio-Rad) and results were expressed as a mean fluorescence intensity (MFI). Blank beads were used in all assays to measure non-specific binding. Any binding to blank beads was subtracted from antigen binding for that sample. Inter-assay variation was tracked by serial dilution of HCMV hyperimmunoglobulin (Cytogam) in Levy-Jennings curves to compare to historical data. Samples were considered positive if they surpassed our positive sample cutoff of 100 MFI, ≥100 beads were counted per sample, and the coefficient of variation per sample duplicate was ≤20%. Black lines denote median values.

gB proteins AD-1 (myBiosource, AA 549–645), Domain I (produced in-house, AA 133–343, and both the 3’ avidin/polyhistidine tags omitted due to hypothesized steric hindrance) and Domain II (produced in-house, AA 112–438 + 343–438, joined with the flexible linker Ile-Ala-Gly-Ser-Gly,). All gB proteins were based on Towne strain gB (genotype 1). gB peptides AD-2 Site 1 (biotin-NETIYNTTLKYGD) and AD-2 Site 2 (biotin-AHSRSGSVQRVTSS). The AD3/MPER domain was determined based on the antibody binding FL ΔTM, but not ectodomain based on ELISA.

### Deglycosylation of gB ectodomain and production of gB glycan mutants

Glycoprotein B ectodomain was deglycosylated according to the New England Biolabs PNGase F Protocol. 18 µg of gB ectodomain was mixed to 2 µL of GlycoBuffer 2 and 5 µL PNGase F, then incubated at 37 °C for 24 h. The deglycosylated protein was then analyzed by Western blot and Coomassie staining (Supplementary Fig. 1). These proteins were then used in an IgG binding ELISA as described above.

Single amino acid mutations were made in gB ectocomain genotype 1 using a QuikChange Lightning Multi Site-Directed Mutagenesis Kit (Agilent Technologies) according to manufacturer protocol. Mutation sequence was confirmed with Sanger sequencing.

### Coomassie and western blot

4–12% Bis-Tris gels were loaded with 10 µl of precision plus protein kaleidoscope prestained protein standard from BioRad and 2 ug of protein in NuPAGE LDS 4X sample buffer (ThermoFisherScientific) per well. Proteins were run in duplicate. One gel was stained with Coomassie. The second gel was transferred using an iBlot 2 gel transfer device. The membrane was blocked for 1 h with 1X PBS/1% Casein blocker. Primary antibody (day 140 serum from gB protein vaccinated rabbit) was added to the blocking buffer and incubated overnight at 4°. Next day the membrane was washed 3X with 1XPBS with 0.1% Tween-20. Secondary antibody goat anti-rabbit IgG-AP conjugate (Sigma Cat. No.AP132A) at a 1:1000 dilution was added and incubated 1 h at room temperature. The membrane was washed 3X with 1XPBS with 0.1% Tween-20. Antibody binding was detected using western blue stabilized substrate for alkaline phosphatase. Gel and membrane were imaged with the molecular imager gel doc XR+ system with image lab software. All blots and gels derive from the same experiment and they were processed in parallel.

### Assessment of gB-specific mAb avidity via surface plasmon resonance (SPR)

Postfusion trimeric gB full lengthΔTM was captured on an NTA sensor chip to ~500 response units (RUs) per cycle using a Biacore X100 (GE Healthcare). The chip was doubly regenerated using 0.35 M EDTA and 0.1 M NaOH followed by 0.5 mM NiCl_2_. Three samples containing only buffer were injected over both ligand and reference flow cells, followed by single injections of each mAb at a concentration of 25 nM. Samples that did not initially result in interpretable sensorgrams were repeated using a concentration of 250 nM. The resulting data were double-reference subtracted and fit to a 1:1 binding model using the Biacore X100 Evaluation software.

### gB-transfected cell IgG binding assay

HEK293T cells were cultured in a T75 flask to ~50% confluency, then transfected using the Effectene Transfection Reagent kit (Qiagen). DNA plasmids expressing GFP (G-C-GFP-W Mega, gift of M. Blasi, Duke University) and Towne strain gB open reading frames were co-transfected. Transfected cells were incubated at 37 °C and 5% CO_2_, washed with PBS once, and detached using TrypLE (Gibco). Cells were re-suspended in HEK293T cell media (DMEM, 10% FBS, 2.5% HEPES, 1.2% Pen-Strep) and assessed for cell count and viability on a Countess Automated Cell Counter (ThermoFisher). Cells were plated in 96-well V-bottom plates (Corning), then centrifuged at 1200 x g for 5 min. Cell supernatant was discarded, and cells were re-suspended in mAbs diluted to a concentration of 5 μg/mL in HEK293T media. Cells and antibodies co-incubated for 2 h at 37 °C and 5% CO_2_. Cells were washed in wash buffer (DPBS + 1% FBS) and re-suspended in LIVE/DEAD far-red infrared cell stain (ThermoFisher Scientific Cat. No.L34973), diluted 1:1000 for 20 min at room temperature. Cells were washed then re-suspended with PE-conjugated mouse anti-human IgG Fc (Southern Biotech Cat. No.9054-09) diluted 1:200 for 25 min at 4 °C. Cells were washed and fixed with 10% formalin for 15 min. Cells were washed then re-suspended in PBS and acquired the following day on an LSR II (BD Biosciences). Percent binding to transfected cells was defined as the PE + GFP+ population of live cells and reported as an average for each sample run in duplicate. Positivity cutoff was determined by PBS control (stained cells without antibody added). A titration of Cytogam was run in each assay to control for inter-assay variability.

### Antibody dependent cellular phagocytosis (ADCP) of whole HCMV virion

Approximately 6 × 10^6^ PFU of concentrated, sucrose gradient-purified AD169r-GFP virus was transferred to a 100,000 kDa Amicon filter (Millipore), then buffer exchanged with 1X PBS, concentrated down to approximately 100 µL, and transferred to a microcentrifuge tube. Next, the AF647 NHS ester (Invitrogen) was reconstituted in DMSO and 10 μg were added to the concentrated, purified virus for direct fluorescent conjugation. This virus-fluorophore mixture incubated at room temperature for 1 h with constant agitation. This virus-enzyme reaction was quenched after 1 h with 80 µL of 1 M Tris-HCl, pH 8.0, then diluted 25x in wash buffer (PBS + 0.1% FBS). Antibodies were diluted to 0.1 mg/mL in wash buffer, 10 µL of which were added to 10 µL fluorophore-conjugated virus in a round bottom, 96-well plate. Plates incubated for 2 h at 37 °C. After incubation, 50,000 THP-1 cells were added to each well of sera and virus. Plates were centrifuged at 1200 x g at 4 °C for a 1-h spinoculation step, then incubated for the subsequent hour at 37 °C to allow ADCP to occur. After incubation, cells were re-suspended and transferred to 96-well V-bottom plates, washed once, stained with LIVE/DEAD Fixable Aqua dead cell stain kit (ThermoFisher Cat. No.L34966), washed once more, then fixed with 10% formalin for 15 min. Cells were spun again and re-suspended in PBS before acquiring events on an LSR II machine (BD Biosciences). The % AF647 + cells were calculated from the full THP-1 population and reported for each sample in duplicate. Gating was set according to setting >98% AF647 + signal from the non-specific antibody control. Inter-assay variability is determined by running a titration of cytogam. 3–74 mAb from the natural infection gB mAb cohort^22^ had ADCP activity and was included as a positive control.

### Reporting summary

Further information on research design is available in the Nature Research Reporting Summary linked to this article.

### Supplementary information

Supplementary Information

Reporting Summary

suplementary data set 1

## Data Availability

All data generated or analyzed during this study are included in this article (and its supplementary files). The detailed study design of phase I clinical trial was previously published^21^. The full trial protocol can be accessed by submitting an inquiry to Merck & Co. via the website: http://engagezone.msd.com/doc/ProcedureAccessClinicalTrialData.pdf.
