# Supplementary material for: Vaccination with a replication-defective cytomegalovirus vaccine elicits a glycoprotein B-specific monoclonal antibody repertoire distinct from natural infection
Source: NPJ Vaccines. 2023 Oct 10;8:154. doi: 10.1038/s41541-023-00749-0 (PMC10564777; doi:10.1038/s41541-023-00749-0)

Supplemental table 1.

mAb Immunogenetics V-160 vaccine

Monoclonal Antibody	Vaccinee	Neutralization IC50 (µg/mL)*	Epitope Specificity	Full Length gBΔTM binding EC50 (µg/ml)	gB Ectodomain binding EC50 (µg/ml)	K_d (nM)	Binding cell associated gB (% GFP positive)
6-151	1	561.3 Complement Dep	None	0.0019	0.0053	<1.3	31.2
6-165	1	ND	None	Non binding	0.094	<1.3	23.7
6-171	1	ND	None	0.0032	0.012	<1.1	26.1
6-172	1	ND	None	0.025	0.044	<2.0	26.7
6-175	1	362.6 Complement Dep	None	0.0052	0.0097	<2.0	28.6
7-408	2	ND	None	0.0017	0.0072	<2.3	30.4
7-409	2	ND	None	0.0059	0.043	<1.6	21.7
7-417	2	ND	None	0.0034	0.0089	<1.1	29.4
7-432	2	ND	None	0.025	0.052	<1.4	19.8
7-459	2	ND	None	0.0021	0.0084	1.7	29.2
7-461	2	ND	None	0.0032	0.019	1.2	27.5
9-845	4	3862.4	None	0.00085	0.0018	2.4	28.6
9-858	4	404.2 Complement Dep	None	0.0015	0.0024	<0.4	29.8
9-862	4	467.5	None	0.0017	0.0037	<1.6	30.5
9-936	4	ND	None	0.029	0.026	1.8	25
9-955	4	ND	None	0.0013	0.0014	<0.5	23.8
9-957	4	ND	None	0.0025	0.0037	<0.6	26.4
12-871	5	ND	AD3/MPER	0.0019	Non binding	14.1	19.1
12-874	5	2878.8 Complement Dep	AD3/MPER	0.0038	0.15	4.7	25.6
12-892	5	16.6 Complement Dep	None	0.0063	0.0023	0.8	24.5
12-959	5	ND	None	0.0017	0.0039	<1.6	27.4
13-928	6	ND	None	0.044	0.015	<1.1	23.9
13-929	6	ND	AD3/MPER	Non binding	Non binding	ND	23.2
13-930	6	ND	None	0.1	0.022	1	24.4
13-980	6	ND	None	0.016	0.034	<2.2	25.7
13-1016	6	ND	AD3/MPER	0.002	Non binding	22.2	13.2
13-1026	6	ND	None	0.0055	0.013	4.4	18.7
13-1034	6	ND	None	0.01	0.016	<2.2	24.8
13-1058	6	ND	None	0.0015	0.0028	<0.3	24.8

- *Neutralization is described in (Li et al 2021).
- One individual had strong serum binding to gB, but gB monoclonal antibodies were not recovered from this vaccinee. (Li et al 2021).
- Li, L. *et al.* A conditionally replication-defective cytomegalovirus vaccine elicits potent and diverse functional monoclonal antibodies in a phase I clinical trial. *npj Vaccines* **6**, 79–93 (2021).

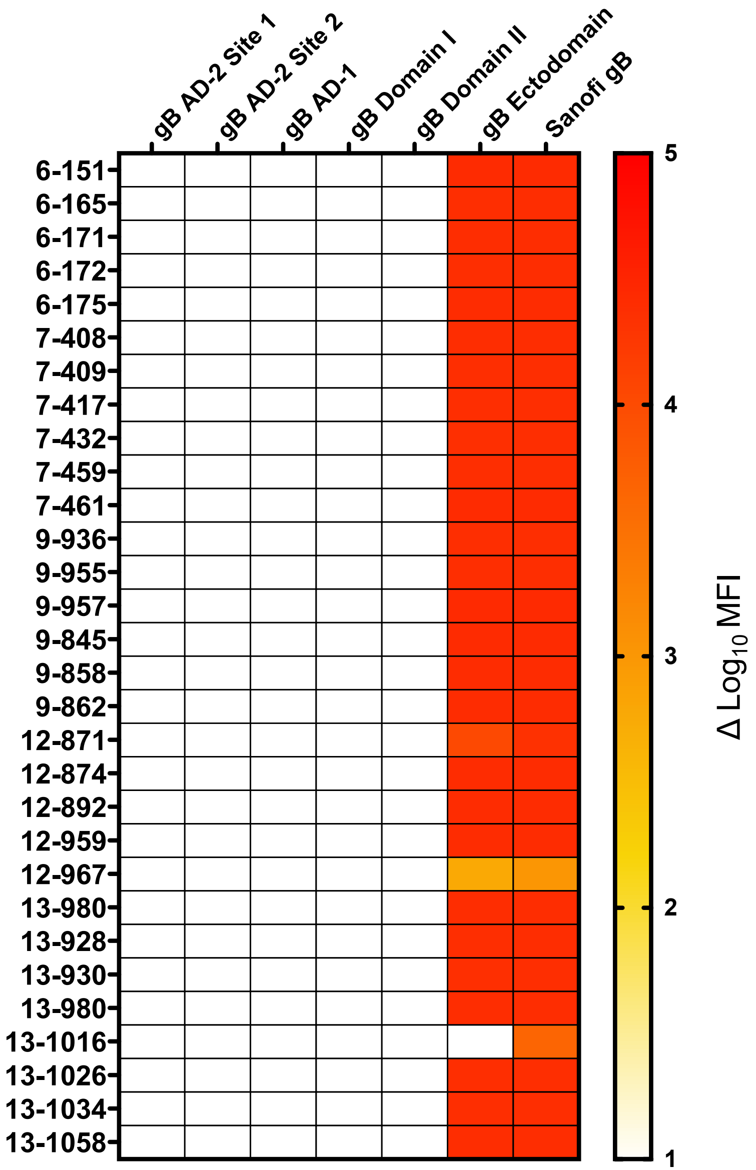

Supplemental Figure 1. V160 vaccinated individuals gB domain-specific mAb binding determined by BAMA. Heat map of gB domain-specific mAb binding strength for gB-specific mAbs represented as log_{10} mean fluorescent intensity (MFI).

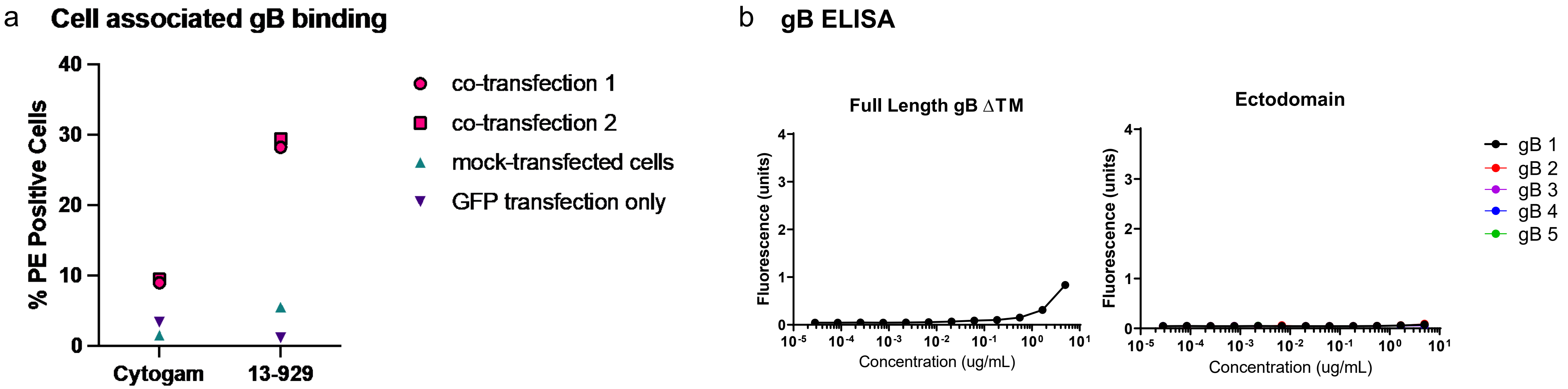

Supplemental Figure 2. A) Plot of mAb 13-929 and cytogam binding to cell-associated gB (cells were co-transfected with gB1 plasmid and GFP), binding to mock-transfected cells (cells treated with all transfection reagents but no plasmid), and binding to cells that were transfected with GFP only. B) 13-929 bound poorly to FLgBΔTM by ELISA. 13-929 did not bind to gB ectodomain genotype 1-5 by ELISA.

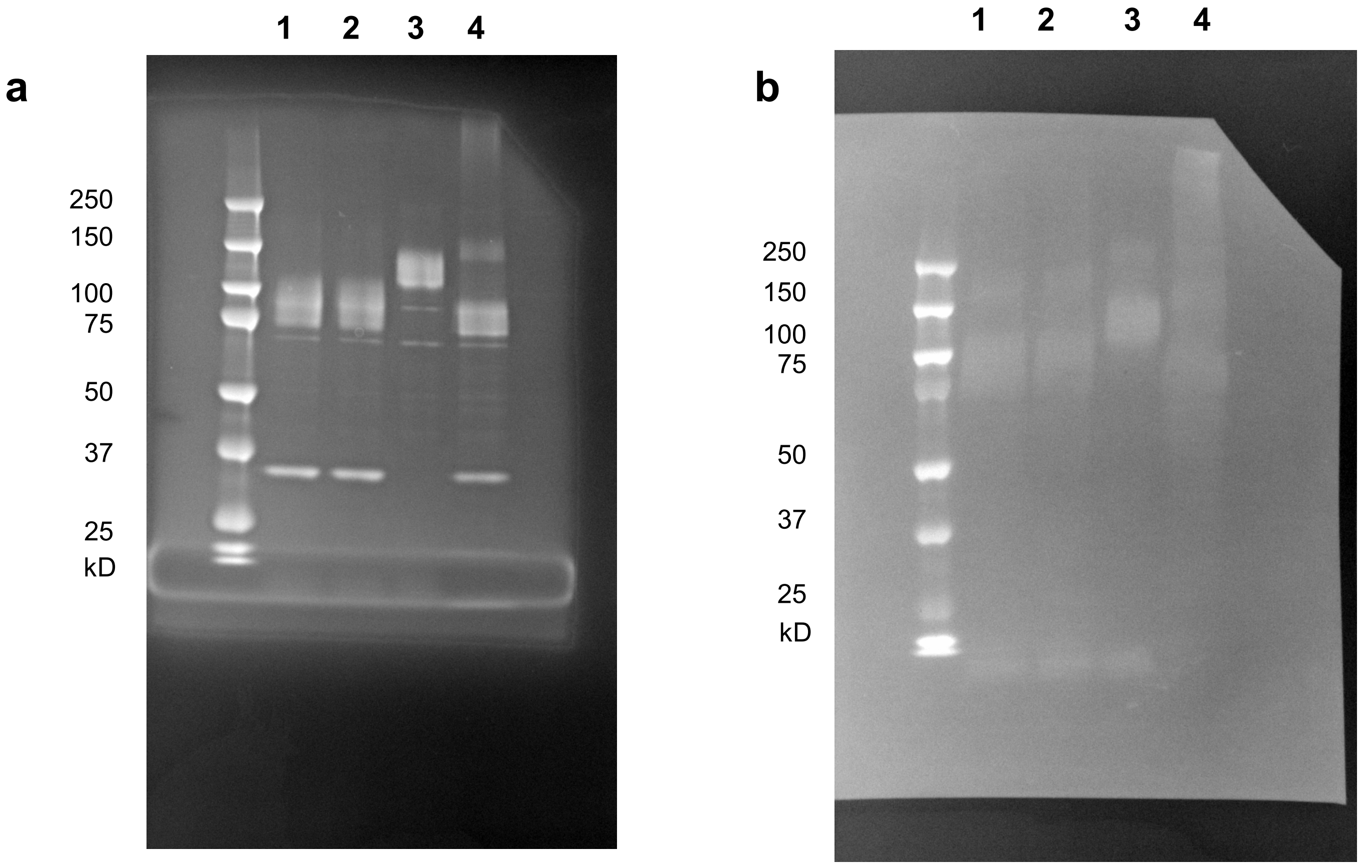

Supplemental Figure 3. A) Coomassie gel of gB ectodomain. Lane 1 and 2 have been treated with PNGase F and lane 3 is untreated protein. Lane 4 is also a gB treated with PNGase F, but not used in assays. The small band in lane 1 and 2 is PNGase F. B) Western blot using day 140 serum from a gB protein vaccinated rabbit.

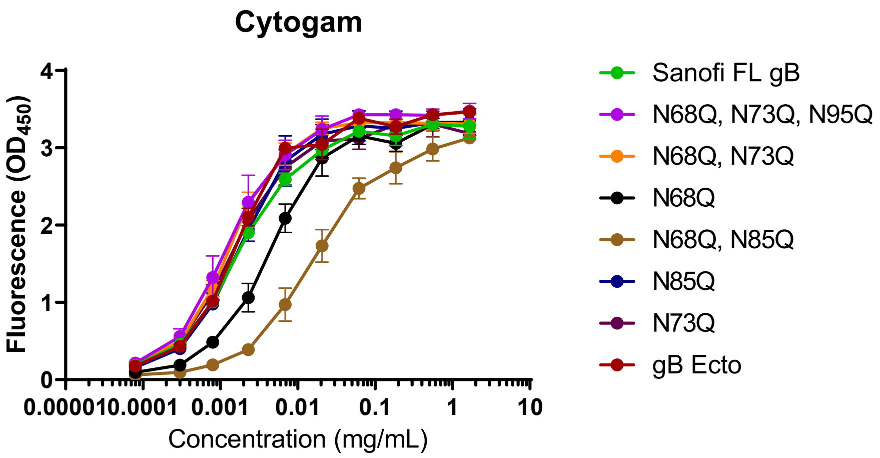

Supplemental Figure 4. Cytogam bound to all gB ectodomain glycan mutants. Cytogam binding to N68Q and (N68Q, N85Q) was slightly impaired compared to all other gB ectodomain glycan mutants.

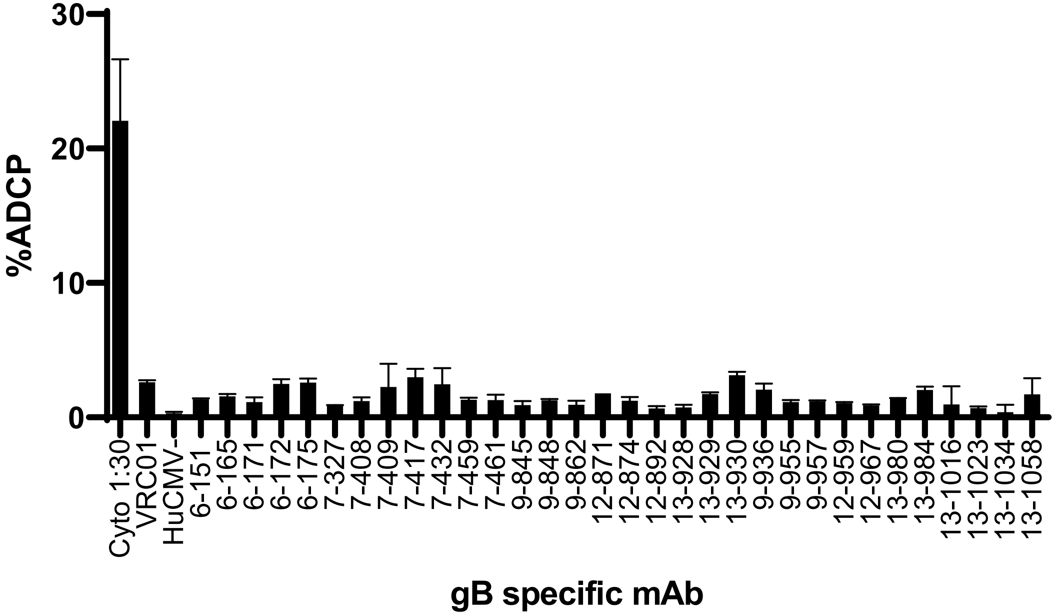

Supplemental figure 5. None of the V-160 gB mAbs showed measurable ADCP activity. mAbs were tested at a concentration of 0.1 mg/ml. There is no measurable antibody-dependent cellular phagocytosis (ADCP) activity detectable for all gB monoclonal antibodies isolated from V-160 vaccinated individuals. Replicates are shown with Mean and Error SD error bars. A Cytogam titration was run as a positive control. Cytogam at a 1:30 dilution is shown on the graph. The negative controls were VRC01 a mAb specific for HIV and Human sera that was known to be CMV negative.

1:6250 Cytogam

gB transfected cell binding gating. Figure 3

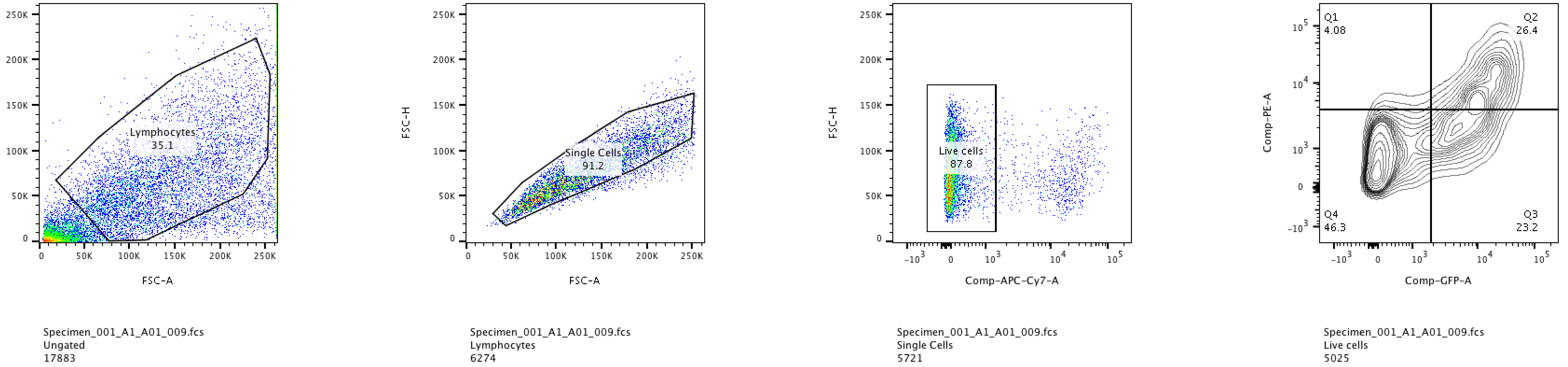

1:6250 Seronegative

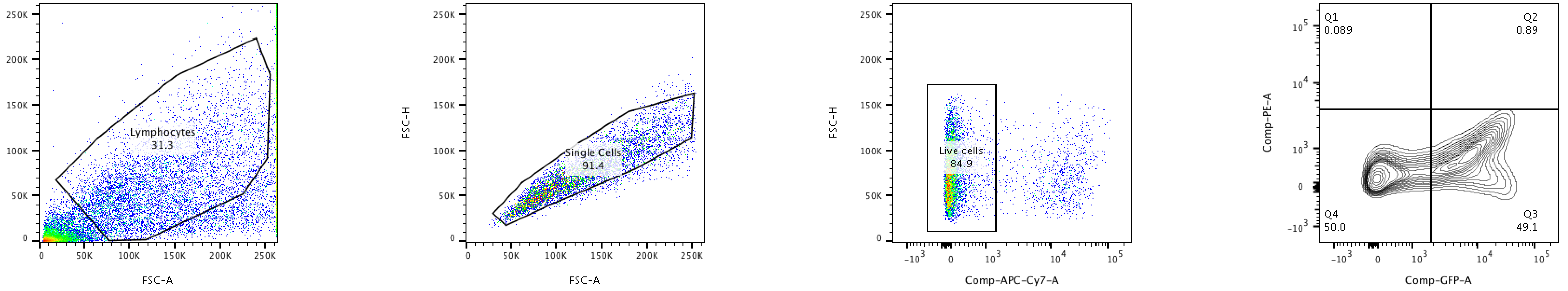

ADCP gating showing positive control Supplemental Figure 5

1:30 cytogam rep 1

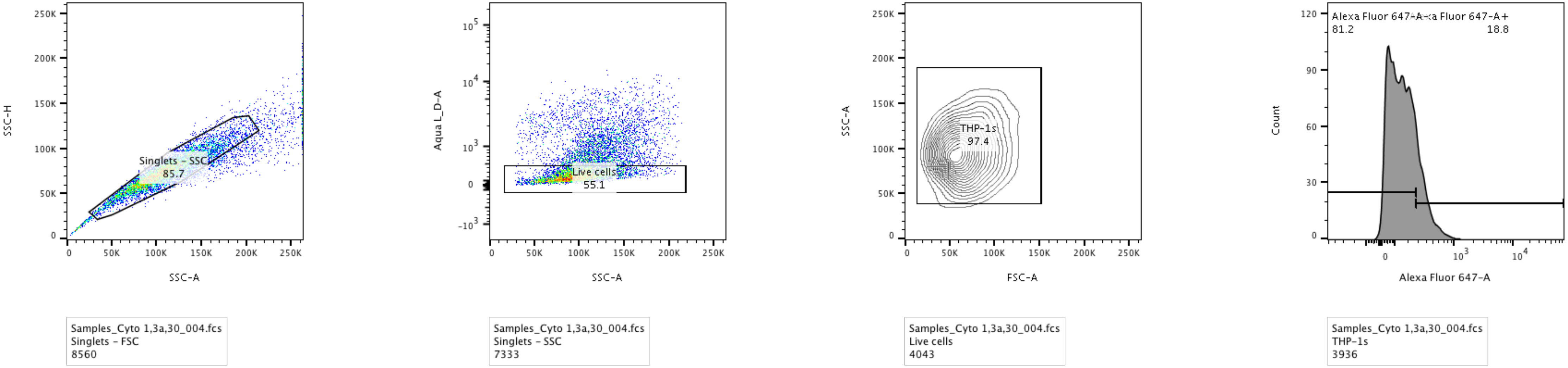

1:30 cytogam rep 2

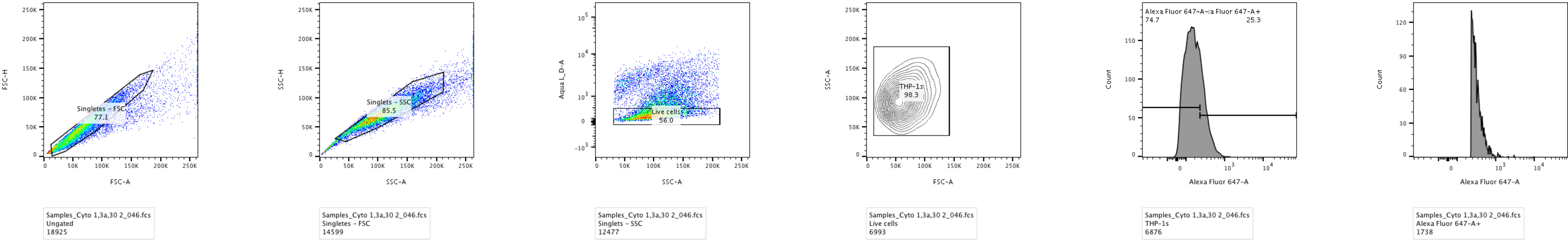

ADCP gating showing negative control Supplemental Figure 5

VRC01 (gating based on this) rep 1

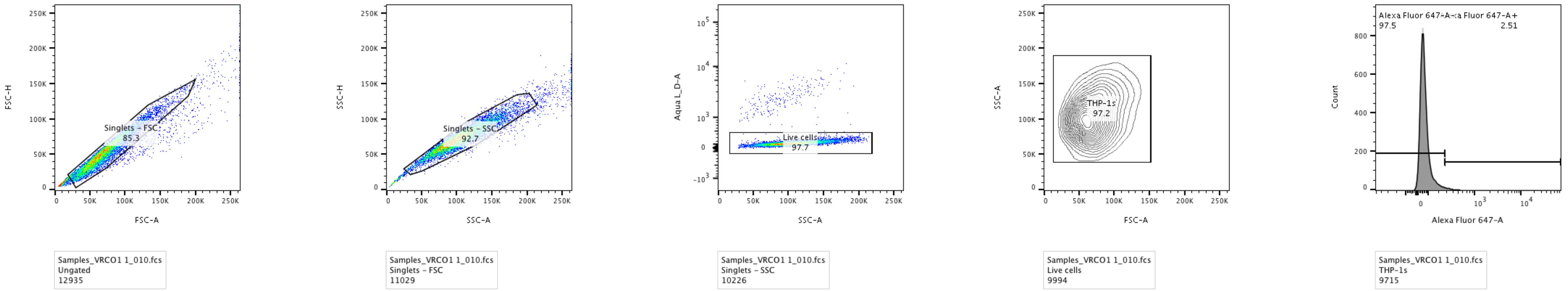

VRC01 (gating based on this) rep 2

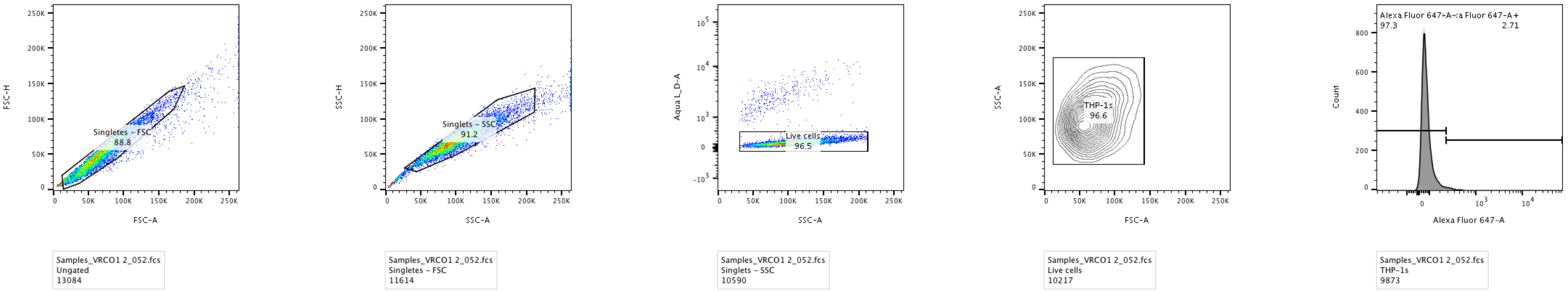

Supplement: Supplementary file 1 — Supplementary Information [file 41541_2023_749_MOESM1_ESM.pdf]
