# Supplementary material for: Vaccination with a replication-defective cytomegalovirus vaccine elicits a glycoprotein B-specific monoclonal antibody repertoire distinct from natural infection
Source: NPJ Vaccines. 2023 Oct 10;8:154. doi: 10.1038/s41541-023-00749-0 (PMC10564777; doi:10.1038/s41541-023-00749-0)
Supplement: Supplementary file 2 — Reporting Summary [file 41541_2023_749_MOESM2_ESM.pdf]

nature portfolio

Corresponding author(s): Sallie R Permar

Last updated by author(s): 8/2/2023

Reporting Summary

Nature Portfolio wishes to improve the reproducibility of the work that we publish. This form provides structure for consistency and transparency in reporting. For further information on Nature Portfolio policies, see our Editorial Policies and the Editorial Policy Checklist.

Statistics

For all statistical analyses, confirm that the following items are present in the figure legend, table legend, main text, or Methods section.

n/a	Confirmed	
☒	☐	The exact sample size (n) for each experimental group/condition, given as a discrete number and unit of measurement
☒	☐	A statement on whether measurements were taken from distinct samples or whether the same sample was measured repeatedly
☒	☐	The statistical test(s) used AND whether they are one- or two-sided<br>*Only common tests should be described solely by name; describe more complex techniques in the Methods section.*
☒	☐	A description of all covariates tested
☒	☐	A description of any assumptions or corrections, such as tests of normality and adjustment for multiple comparisons
☒	☐	A full description of the statistical parameters including central tendency (e.g. means) or other basic estimates (e.g. regression coefficient) AND variation (e.g. standard deviation) or associated estimates of uncertainty (e.g. confidence intervals)
☒	☐	For null hypothesis testing, the test statistic (e.g. F, t, r) with confidence intervals, effect sizes, degrees of freedom and P value noted<br>*Give P values as exact values whenever suitable.*
☒	☐	For Bayesian analysis, information on the choice of priors and Markov chain Monte Carlo settings
☒	☐	For hierarchical and complex designs, identification of the appropriate level for tests and full reporting of outcomes
☒	☐	Estimates of effect sizes (e.g. Cohen's d, Pearson's r), indicating how they were calculated

Our web collection on statistics for biologists contains articles on many of the points above.

Software and code

Policy information about availability of computer code

Data collection	Spectramax plate reader (Thermo Scientific), Bio-Plex 200 (Bio-Rad), Biacore X100 Evaluation software, FACSDiva
Data analysis	Graph Pad Prism, Flow Jo, Excel

For manuscripts utilizing custom algorithms or software that are central to the research but not yet described in published literature, software must be made available to editors and reviewers. We strongly encourage code deposition in a community repository (e.g. GitHub). See the Nature Portfolio guidelines for submitting code & software for further information.

Data

Policy information about availability of data

All manuscripts must include a data availability statement. This statement should provide the following information, where applicable:

- Accession codes, unique identifiers, or web links for publicly available datasets
- A description of any restrictions on data availability
- For clinical datasets or third party data, please ensure that the statement adheres to our policy

Data Availability
All data generated or analyzed during this study are included in this article (and its supplementary files). The detailed study design of phase I clinical trial was previously published21. The full trial protocol can be accessed by submitting an enquiry to Merck & Co. via the website: http://engagezone.msd.com/doc/

ProcedureAccessClinicalTrialData.pdf.

Research involving human participants, their data, or biological material

Policy information about studies with human participants or human data. See also policy information about sex, gender (identity/presentation), and sexual orientation and race, ethnicity and racism.

Reporting on sex and gender	*Use the terms sex (biological attribute) and gender (shaped by social and cultural circumstances) carefully in order to avoid confusing both terms. Indicate if findings apply to only one sex or gender; describe whether sex and gender were considered in study design; whether sex and/or gender was determined based on self-reporting or assigned and methods used. Provide in the source data disaggregated sex and gender data, where this information has been collected, and if consent has been obtained for sharing of individual-level data; provide overall numbers in this Reporting Summary. Please state if this information has not been collected. Report sex- and gender-based analyses where performed, justify reasons for lack of sex- and gender-based analysis.*
Reporting on race, ethnicity, or other socially relevant groupings	*Please specify the socially constructed or socially relevant categorization variable(s) used in your manuscript and explain why they were used. Please note that such variables should not be used as proxies for other socially constructed/relevant variables (for example, race or ethnicity should not be used as a proxy for socioeconomic status). Provide clear definitions of the relevant terms used, how they were provided (by the participants/respondents, the researchers, or third parties), and the method(s) used to classify people into the different categories (e.g. self-report, census or administrative data, social media data, etc.) Please provide details about how you controlled for confounding variables in your analyses.*
Population characteristics	*Describe the covariate-relevant population characteristics of the human research participants (e.g. age, genotypic information, past and current diagnosis and treatment categories). If you filled out the behavioural & social sciences study design questions and have nothing to add here, write "See above."*
Recruitment	*Describe how participants were recruited. Outline any potential self-selection bias or other biases that may be present and how these are likely to impact results.*
Ethics oversight	*Identify the organization(s) that approved the study protocol.*

Note that full information on the approval of the study protocol must also be provided in the manuscript.

Field-specific reporting

Please select the one below that is the best fit for your research. If you are not sure, read the appropriate sections before making your selection.

☒ Life sciences ☐ Behavioural & social sciences ☐ Ecological, evolutionary & environmental sciences

For a reference copy of the document with all sections, see nature.com/documents/nr-reporting-summary-flat.pdf

Life sciences study design

All studies must disclose on these points even when the disclosure is negative.

Sample size	gB monoclonal antibodies isolated from 6 V-160 vaccinated individuals
Data exclusions	If a monoclonal antibody did not bind to a form of gB it was excluded from this study.
Replication	We run positive controls monitored by Levy-Jennings curves to validate consistency within 3 standard deviation between each experiment. Within each experiment samples are run at minimum in duplicate and depending on the nature of the assay a specific CV is required for data to be included. If replicates are outside of range that data point is repeated.
Randomization	All V-160 gB mAbs were run at the same time. mAbs from natural infected individuals were run in a previous study together as a cohort. There are two assays, gB genotype and glycosylation that were new to this study and both V-160 and natural infection gB mAbs were run at the same time in these assays.
Blinding	All mAbs were run at the same time.

Reporting for specific materials, systems and methods

We require information from authors about some types of materials, experimental systems and methods used in many studies. Here, indicate whether each material, system or method listed is relevant to your study. If you are not sure if a list item applies to your research, read the appropriate section before selecting a response.

Materials & experimental systems

n/a	Involved in the study	
☐	☒	Antibodies
☐	☒	Eukaryotic cell lines
☒	☐	Palaeontology and archaeology
☒	☐	Animals and other organisms
☒	☐	Clinical data
☒	☐	Dual use research of concern
☒	☐	Plants

Methods

n/a	Involved in the study	
☒	☐	ChIP-seq
☐	☒	Flow cytometry
☒	☐	MRI-based neuroimaging

Antibodies

Antibodies used: Cytogam (Cytomegalovirus immune globulin) is commercially available from a clinical source, VRC01 is a HIV mAb isolated by NIH VRC, and is available in the AIDS reagent program, Rabbit serum from gB vaccinated rabbit, from a study in S.R.P laboratory, this stock contains limited quantity, but other rabbit serums are commercially available.

Validation: Both Cytogam and VRC 01 are antibodies used in clinic and come from controlled sources. Cytogam was always positive for gB binding and VRC 01 never bound to gB. The Rabbit serum from gB vaccinated rabbits bound to a purified gB protein validating that there are antibodies to gB present in this serum.

Eukaryotic cell lines

Policy information about cell lines and Sex and Gender in Research

Cell line source(s): HEK293T, THP-1 both cell lines are from ATCC

Authentication: None

Mycoplasma contamination: Cell lines were negative for mycoplasma contamination

Commonly misidentified lines (See ICLAC register): *Name any commonly misidentified cell lines used in the study and provide a rationale for their use.*

Flow Cytometry

Plots

Confirm that:

☒ The axis labels state the marker and fluorochrome used (e.g. CD4-FITC).

☒ The axis scales are clearly visible. Include numbers along axes only for bottom left plot of group (a 'group' is an analysis of identical markers).

☒ All plots are contour plots with outliers or pseudocolor plots.

☒ A numerical value for number of cells or percentage (with statistics) is provided.

Methodology

Sample preparation: HEK293T were used for cell associated gB assay details in methods, THP-1 were used for ADCP details in methods

Instrument: BD LSRII Cell analyzer with 4 lasers, 488 nm, 532 nm, 642 nm, and 405 nm

Software: Diva was used on instrument and Flow Jo was used to analyze data

Cell population abundance: Cell populations are noted in representative gating included in supplemental files

Gating strategy: Gating strategy is noted on supplemental file

☒ Tick this box to confirm that a figure exemplifying the gating strategy is provided in the Supplementary Information.
